# Application value of hand-sewn anastomosis in totally laparoscopic total gastrectomy for gastric cancer

**DOI:** 10.1186/s12957-021-02249-8

**Published:** 2021-08-04

**Authors:** Zeshen Wang, Yuzhe Wei, Xirui Liu, Zhenglong Li, Guanyu Zhu, Yanfeng Li, Kuan Wang

**Affiliations:** Department of Gastrointestinal Surgery, Harbin Medical University Cancer Hospital, No.150, Haping Road, Nangang District, Harbin, 150086 Heilongjiang China

**Keywords:** Gastric cancer; Anastomosis, surgical; gastric surgery, suture techniques, digestive system surgical procedures

## Abstract

**Background:**

Digestive tract reconstruction in totally laparoscopic total gastrectomy can be divided into two types: instrument anastomosis and hand-sewn anastomosis. This study explored the feasibility and safety of hand-sewn sutures in esophagojejunostomy of totally laparoscopic total gastrectomy, compared with instrument anastomosis using an overlap linear cutter.

**Methods:**

This retrospective cohort study was conducted from January 2017 to January 2020 at one institution. The clinical data of 50 patients who underwent totally laparoscopic total gastrectomy, with an average follow-up time of 12 months, were collected. The clinicopathologic data, short-term survival prognosis, and results of patients in the hand-sewn anastomosis (*n*=20) and the overlap anastomosis (*n*=30) groups were analyzed.

**Results:**

There were no significant differences between the groups in sex, age, body mass index, American Society of Anesthesiologists score, tumor location, preoperative complications, abdominal operation history, tumor size, pTNM stage, blood loss, first postoperative liquid diet, exhaust time, or postoperative length of hospital stay. The hand-sewn anastomosis group had a significantly prolonged operation time (204±26.72min versus 190±20.90min, *p*=0.04) and anastomosis time (58±22.0min versus 46±15.97min, *p*=0.029), and a decreased operation cost (CNY 77,100±1700 versus CNY 71,900±1300, *p*<0.0001). Postoperative complications (dynamic ileus, abdominal infection, and pancreatic leakage) occurred in three patients (15%) in the hand-sewn anastomosis group and in four patients (13.3%) in the overlap anastomosis group (anastomotic leakage, anastomotic bleeding, dynamic ileus, and duodenal stump leakage).

**Conclusion:**

The hand-sewn anastomosis method of esophagojejunostomy under totally laparoscopic total gastrectomy is safe and feasible and is an important supplement to linear and circular stapler anastomosis. It may be more convenient regarding obesity, a relatively high position of the anastomosis, edema of the esophageal wall, and short jejunal mesentery.

## Background

Since Kitano et al. [1] first reported laparoscopic-assisted Billroth I gastrectomy for early-stage gastric cancer in 1994, laparoscopic distal gastrectomy (LDG) has been accepted as a technically and oncologically feasible method [2–5]. However, there is no uniform standard for totally laparoscopic total gastrectomy (TLTG) due to the difficulty in digestive tract reconstruction and the diversity of anastomosis methods [6–12]. TLTG digestive tract reconstruction can be divided into two categories: instrument anastomosis, which mainly employs a circular anastomosis technique and linear cutting, and hand-sewn anastomosis. At present, instrument anastomosis is the mainstay procedure, which provides convenience for surgeons; however, it cannot avoid the problems associated with anvil implantation, uncertainty of the cutting edge, and high cost in special circumstances [7, 13]. Therefore, researchers began to explore hand-sewn anastomosis performed using a laparoscope. Although hand-sewn anastomosis has more requirements for the operation and a relative longer anastomosis time, its advantages include a good surgical field of view, operation in a narrow space, avoiding excessive traction, and ease in obtaining the pathology of the esophageal cutting edge before anastomosis. Notably, hand-sewn anastomosis has been reported to be a safe and feasible procedure [6–9, 13–15], although clinical research remains insufficient. Therefore, we recorded the number of patients who underwent TLTG with hand-sewn anastomosis and with overlap anastomosis from January 2017 to January 2020. We then compared the clinical results of the two groups, aiming to explore the application prospects of hand-sewn anastomosis in the evaluation of TLTG.

## Materials and methods

### Patients

Clinical and pathological data of 50 patients who underwent TLTG in the Third Affiliated Hospital of Harbin Medical University from January 2017 to January 2020 were collected.

Inclusion criteria were as follows: (1) preoperative gastroscopy and enhanced CT revealed a gastric tumor and pathological biopsy showed adenocarcinoma; (2) no distant metastasis to the liver, lungs, and so on; (3) the TNM clinical stage was I–III; (4) TLTG was performed by the same group of doctors. Exclusion criteria were as follows: patients with a history of gastric surgery and those with a history of preoperative radiotherapy and chemotherapy.

After applying the exclusion criteria, a total of 50 patients were included in the study. According to different anastomosis methods, they were divided into a hand-sewn anastomosis (*n* = 20) and an overlap (*n* = 30). Due to the retrospective nature of the study, written informed consent was not required.

### Surgical methods

**Fig. 1:**
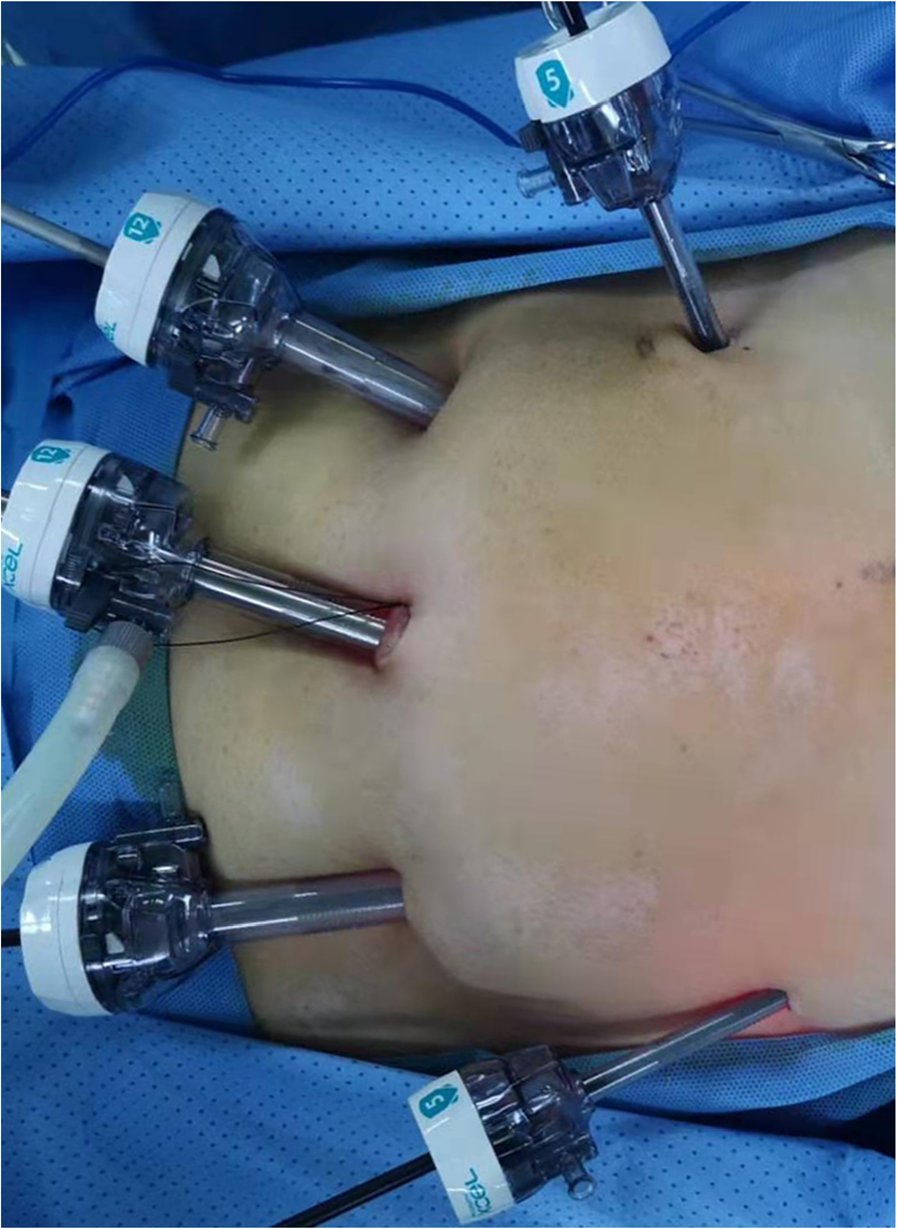
Distribution of trocars

Under general anesthesia, the patient was placed in the supine position, the operator was on the right side of the patient, the assistant was on the left side of the patient, and the mirror holder was on the foot side of the patient. A trocar was placed into the observation port by making a small incision 1 cm above the umbilicus to establish a pneumoperitoneum, and a pressure of 14 mmHg was maintained. No visible peritoneal metastasis or ascites was found by exploration. Under the direct view of the laparoscope, a 12-mm trocar was placed under the costal margins of the left and right anterior axillary lines as the main operation port, and 5-mm trocars were placed on the left and right midclavicular lines parallel to the umbilicus as the auxiliary ports (Fig. 1). Standard D2 lymph node dissection and radical tumor treatment were performed.

### Digestive tract reconstruction

#### Overlap anastomosis

**Fig. 2:**
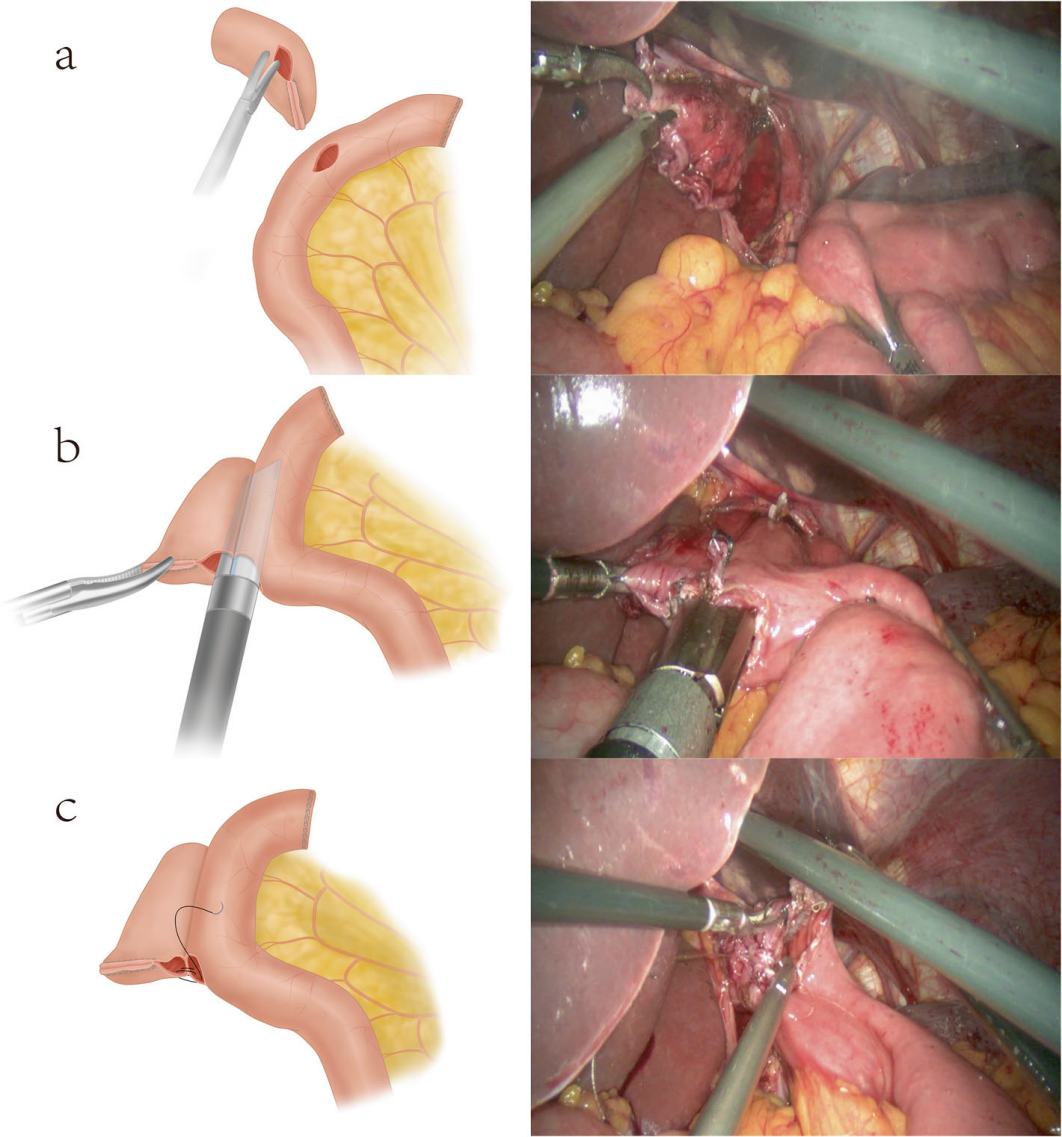
**a** A hole is punched in the right distal esophageal stump, and a small incision is made on the dorsal mesentery 7 cm distal to the anastomosis line in the distal jejunum. **b** Esophageal wall-jejunal side-to-side anastomosis. **c** Hand-sewn suture and closure of the common openings

The abdominal segment of the esophagus was sufficiently dissociated. A hole was created in the right distal esophageal stump; the jejunum was disconnected with a 60-mm stapler that was 15–20 cm away from the ligament of Treitz. A small incision was then made on the dorsal mesentery 7 cm distal to the anastomosis line at the distal jejunum (Fig. 2a). The staple cartridge was on the jejunum side. The other side used gastric tube guidance for entry for a lateral esophageal wall-jejunal side-to-side anastomosis (Fig. 2b). The common openings were closed using hand-sewn anastomosis (Fig. 2c). Jejuno-jejunal side-to-side anastomosis was performed 50 cm distal to the esophagojejunal anastomosis, and the common openings were closed.

#### Hand-sewn anastomosis

**Fig. 3:**
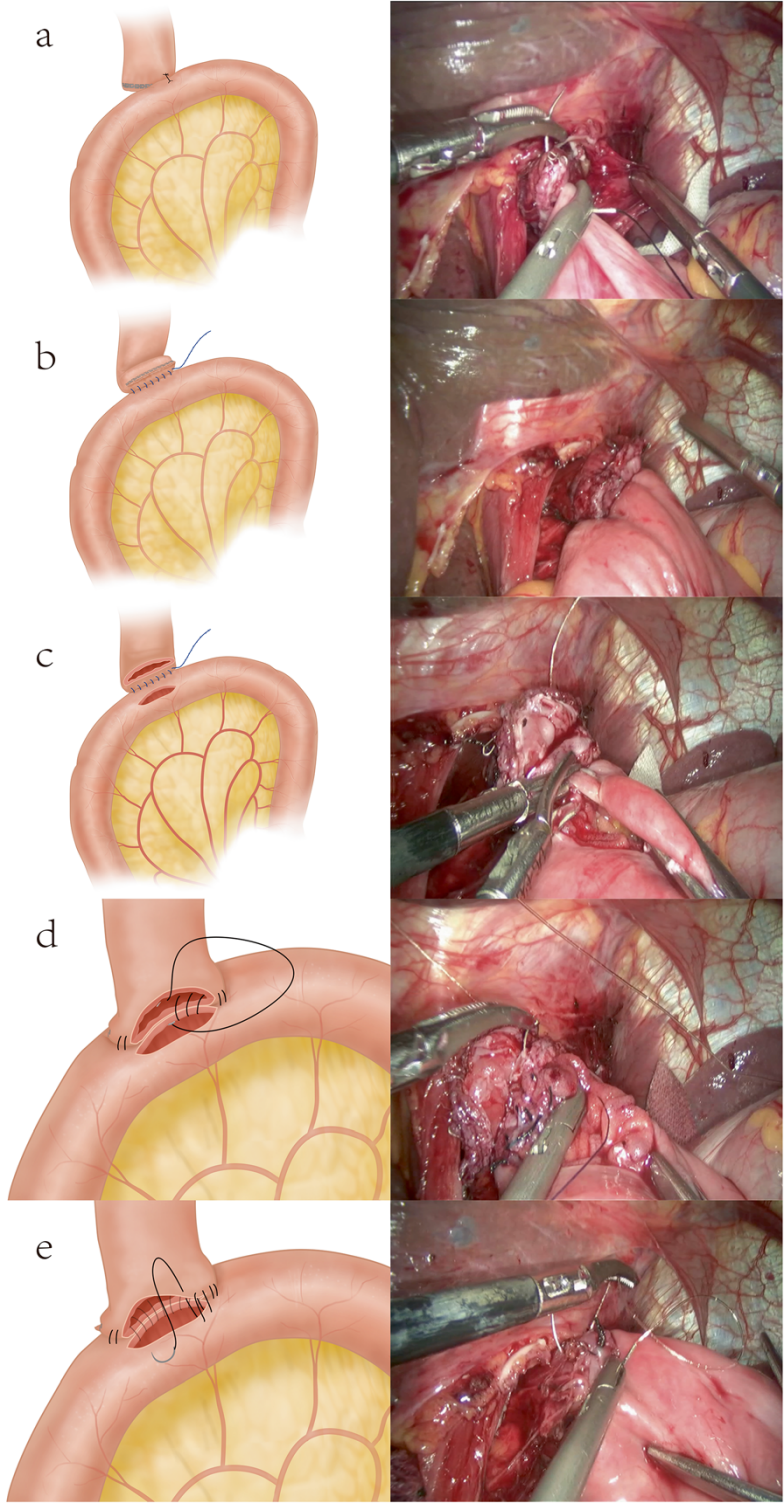
**a** A stitch is made to fix the left esophageal stump with the dorsal mesojejunum. **b** Continuous suturing of the posterior wall of the esophageal stump and the posterior wall of jejunum is performed from right-to-left with a barbed suture, and the barbed sutures are retained. **c** Openings in the esophageal stump and dorsal mesentery. **d** Continuous suture of the posterior wall of the anastomosis. **e** Continuous suture of the anterior wall of the anastomosis

The jejunum was lifted, and a stitch was made to fix the left esophageal stump with the dorsal mesojejunum at a distance of 20–25 cm from the ligament of Treitz (Fig. 3a). Afterwards, continuous suturing of the posterior wall of the esophageal stump and the posterior wall of jejunum was performed from right-to-left with a barbed suture, and the barbed sutures were retained (Fig. 3b). The distal esophageal stump was separated with an ultrasonic scalpel, and a matching incision was made on the dorsal mesentery (Fig. 3c). When suturing the posterior wall, the seromuscular layer was continuously sutured with a barbed suture first; then, the entire posterior wall was intermittently sutured in a right-to-left direction by means of an inverting suture (Fig. 3d). When suturing the anterior wall, the full thickness of the anterior wall was intermittently sutured from left to right with an everting suture. Finally, the seromuscular layer was continuously sutured with a barbed suture (Fig. 3e). Reinforcement suturing was performed at the discontinuity on the periphery of the anastomosis. The posterior jejunum was disconnected, and a jejuno-jejunal side-to-side anastomosis was performed 40 cm away from the anastomosis.

### Postoperative management

**Fig. 4:**
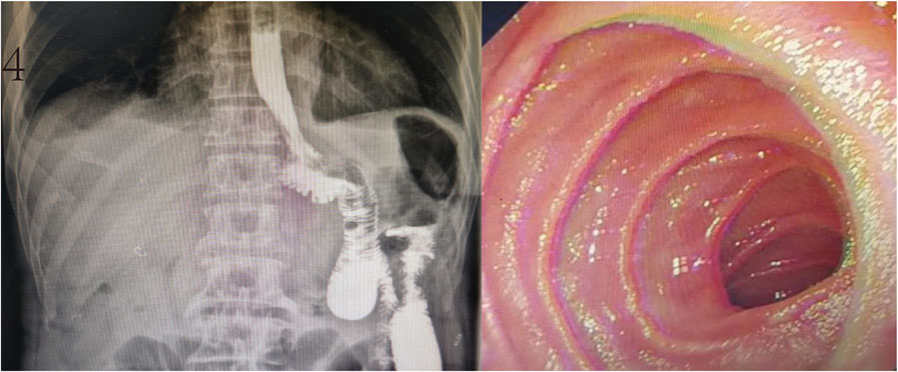
Postoperative adjuvant examination images of a patient in the hand-sewn anastomosis group. **a** Angiography on day five after surgery shows no anastomotic leakage. **b** Electronic gastroscopy at 6 months after surgery shows no anastomotic stenosis

The gastric tube was removed on the first day after surgery, and patients were allowed to ingest a small amount of liquid food several times after the first postoperative exhaust. A CT scan and upper gastrointestinal radiography were performed on day five after surgery to assess whether there was anastomotic leakage (Fig. 4a). The patient was discharged 7–8 days after surgery. Electronic gastroscopy was performed 6 months after discharge to observe whether there was anastomotic stenosis (Fig. 4b).

### Statistical analysis

The SPSS 22.0 statistical software was used to perform the statistical analysis. Measurement data that conformed to a normal distribution were expressed as x±s, and comparison between the groups was performed using an independent samples *t*-test. Intergroup comparison of enumeration data was performed using *χ*^2^ or a continuity-adjusted *χ*^2^ test. *p*<0.05 indicated that the difference was statistically significant.

## Results

The clinicopathologic characteristics of the two groups are shown in Table 1. There were 20 patients in the hand-sewn anastomosis group, including 14 men and 6 women. The mean age was 56.3±7.8 years, and the mean body mass index (BMI) was 24.0±3.0 kg/m^2^. The tumor was located in the fundus or upper part of the stomach in 14 patients, and in the middle part of the stomach in 6 patients. There were 30 patients in the overlap anastomosis group, including 21 men and 9 women. The mean age was 57.1±5.5 years, and the mean BMI was 23.3±3.6 kg/m^2^. The tumor was located in the fundus or upper part of the stomach in 17 patients and in the middle part of the stomach in 13 patients.

**Table 1:** General clinical data of the patients

	Hand-sewn anastomosis group	Overlap anastomosis group	*p* value
Sex			1.000
Male	14 (70)	21 (70)	
Female	6 (30)	9 (30)	
Age (years)	56.3±7.8	57.1±5.5	0.640
Body mass index (kg/m^2^)	24.0±3.0	23.3±3.6	0.525
ASA score			0.815
I	12 (60.0)	17 (56.7)	
II	8 (40.0)	13 (43.3)	
III	0	0	
Tumor location			0.341
Upper part of the stomach	14 (70.0)	17 (56.7)	
Middle part of the stomach	6 (30.0)	13 (43.3)	
Preoperative complications			0.924
0	11 (55.0)	14 (46.7)	
1	6 (30.0)	11 (36.7)	
2	3 (15.0)	5 (16.6)	
≥3	0	0	
History of abdominal surgery	2 (10.0)	5 (16.6)	
Follow-up time (months)	12	12	
TNM staging			0.660
I	3 (15.0)	8 (26.6)	
II	8 (40.0)	11 (36.7)	
III	9 (45.0)	11 (36.7)	
IV	0	0	
Tumor size (cm)	2.45±1.38	3.03±1.82	0.238

Note: Data are expressed as mean±standard deviation or *n* (%)

*ASA* American Society of Anesthesiologists score, *TNM* tumor, node, metaphysis stage

### Intraoperative and postoperative results

The intraoperative and early postoperative results are shown in Table 2. No procedures were converted to laparotomy during the operation. The mean operation time (from placement of the first trocar to closure of the abdominal cavity) was significantly prolonged in the hand-sewn anastomosis group compared with the overlap anastomosis group (204±26.72min versus 190±20.90min, *p*=0.04), and the mean anastomosis time (from the first suture to the end of anastomosis) was significantly prolonged in the hand-sewn anastomosis group compared with the overlap anastomosis group (58±22.0min versus 46±15.97min, *p*=0.029). The operation cost was significantly increased for the overlap anastomosis group compared with the hand-sewn anastomosis group (CNY 77,100±1700 versus CNY 71,900±1300, *p*<0.0001). There were no significant differences in blood loss, time to first liquid diet, time to first postoperative exhaust, postoperative length of hospital stay, total number of dissected lymph nodes, and number of lymph node metastases.

**Table 2:** Operation and postoperative results

	Hand-sewn anastomosis group	Overlap anastomosis group	*p* value
Operation time (min)	204.70±26.72	190.47±20.90	0.04
Anastomosis time (min)	58.70±22.0	46.60±15.97	0.029
Blood loss (ml)	118.00±61.89	116.53±51.39	0.928
Conversion to laparotomy	0	0	
Time to first postoperative liquid diet (days)	4.30±0.92	3.87±0.82	0.088
Time to first postoperative exhaust (days)	3.99±1.00	4.23±0.82	0.277
Postoperative hospital stay (days)	7.90±0.79	7.57±0.77	0.145
Secondary operation	0	0	
Operation cost (CNY 1,000)	71.9±1.7	77.1±1.3	<0.0001

Data are expressed as mean±standard deviation or *n* (%)

### Postoperative complications

The mortality rate within 30 days after operation was 0. The median postoperative follow-up time of the two groups was 12 months. The main focus of follow-up was whether there were anastomotic complications. Postoperative complications occurred in three patients (15%) in the hand-sewn anastomosis group; they were dynamic ileus, abdominal infection, and pancreatic leakage. Postoperative complications occurred in four patients (13.3%) in the overlap anastomosis group and included anastomotic leakage, anastomotic bleeding, dynamic ileus, and duodenal stump leakage.

## Discussion

Previous studies have shown that in the surgical treatment of advanced gastric cancer, laparoscopic treatment is not inferior to open surgery in terms of postoperative biological efficacy [4], which indicates that laparoscopic minimally invasive treatment has entered a new stage of clinical application for advanced gastric cancer. In addition, considering the increasing incidence of adenocarcinomas of the esophagogastric junction globally, the development of laparoscopic total gastrectomy (LTG) within the concept of minimal invasiveness is inevitable. However, the esophagogastric anastomosis in TLTG remains an important clinical challenge [10, 16].

The digestive tract reconstruction for LTG can be divided into two categories, laparoscopic-assisted total gastrectomy (LATG) and TLTG. Compared with TLTG, LATG is closer to the state of laparotomy; however, it is difficult to place the anvil and operate within the assisted small incision at the same time. In addition, the excessive traction of the incision will increase postoperative pain and the risk of anastomotic bleeding [17], which limits its clinical application and minimally invasive effect to a certain extent. In contrast, TLTG is monitored using a laparoscopic high-definition visual field during the entire process; consequently, patients receive improved minimally invasive benefits, there is a wider application value, and good clinical outcomes can be achieved [13, 18–20].

At present, instrument anastomosis remains the mainstream procedure in TLTG digestive tract reconstruction. Furthermore, among the instrument anastomosis techniques, the circular stapler technique does not require the exposure of a large portion of the esophagus, and the blood supply of the anastomosis is sufficient, especially on the side of esophageal stump, which can still be disconnected at a relatively high position. However, the main difficulty lies in the placement of the anvil. Transabdominal insertion is easily affected by factors such as the patient’s body size; transoral insertion may scratch the esophageal mucosa and even the muscle layer [21]. In addition, the large size of the circular stapler, when entering through a small abdominal incision, may cause problems with maintaining the pneumoperitoneum pressure, and imprecision in excitation cannot be avoided.

Compared with circular anastomosis, using a linear stapler for side-to-side anastomosis is relatively convenient, and the incidence of stenosis is low [18, 20]. However, side-to-side anastomosis should not be adopted for patients with anastomosis at a relatively high position, with some even higher than the esophageal hiatus in the diaphragm, because it is inconvenient to operate, and may cause anastomosis of the submucosal dissection with a false lumen in the esophageal wall. In addition, edema may exist in the esophageal wall when the primary focus is obstructed, and the risk of leakage is relatively high after closure.

Therefore, considering instrument anastomosis in TLTG, though they are convenient, efficient and time-saving, they cannot provide a full scope of its advantages and cannot perfectly solve the surgeon’s concerns for certain patients. Hence, hand-sewn suture, among the basic skills of surgeons, can be relevant in laparoscopy today because it not only is convenient for an operation on the incisal margin, but also does not cause tension in the entire suture process. In addition, it does not require exposure of a relatively long esophageal stump, allows improved blood supply, and saves costs. Therefore, hand-sewn suture may be the preferred choice under special circumstances.

In 2011, So et al. [6] first reported hand-sewn esophagojejunal suture under TLTG and preliminarily discussed the advantages and disadvantages of this method. In the present study, although the operation time and anastomosis time were significantly longer than those in the overlap anastomosis group, there was no significant difference in intraoperative complications or early postoperative clinical outcomes. There were no anastomotic complications (anastomotic leakage and anastomotic stenosis) in 20 cases of hand-sewn anastomosis, which were significantly lower than the incidence of anastomotic leakage (0–5%) and anastomotic stenosis (0–4.8%) reported in the literature [7–9, 15, 22, 23] (Table 3). The author thinks that this finding is due to the following points: (1) The hand-sewn method adopted by our team allows us to carefully observe the inside of the anastomosis under laparoscopic monitoring, and effectively prevent the compression of anastomotic bleeding and hematoma. (2) It is more reliable to perform hand-sewn anastomosis of the posterior wall varus and anterior wall valgus, and an intermittent full-thickness suture of the anterior and posterior walls, respectively, combined with continuous sutures of barbed suture thread. In case of anastomotic leakage after overlap anastomosis, there is a large shear force above the anastomotic site, which is not conducive to the recovery of anastomotic stoma. In contrast, hand-sewn anastomosis is easier to recover because of its low tension and even if leakage occurs after operation. (3) The author’s team has many years of experience, and the learning curve completion for laparoscopic distal gastrectomy may improve the safety of the surgery. By contrast, there was one case of anastomotic leakage in the overlap group, which might be because of the relatively high position of the disconnection and slight edema of the esophageal wall. Additionally, there was some difficulty in closing the common openings. After conservative treatment, the anastomotic leakage was improved, and the electronic gastroscopy procedure 6 months after operation revealed no anastomotic stenosis, 1 case of anastomotic bleeding (improved after conservative treatment).

**Table 3:** Situations of postoperative anastomotic leakage and anastomotic stenosis after hand-sewn anastomosis of TLTG in published articles

Author	Year	Total cases	Operation time (min)	Anastomosis time (min)	Anastomotic fistula	Anastomotic stenosis	Anastomotic bleeding
Kwang Oh [6]	2011	6	379.7	81.5	0	0	0
Ke Chen [13]	2016	59	257.4	46.3	0	0	0
Xiaowu Xu [9]	2017	100	267.0	45.0	1%	0	0
Enrique Noreo [8]	2017	51	337.0	NONE	3.9%	1.9%	0
Zhipeng Sun [15]	2019	20	216.5	44.4	5%	5%	0
Jiafei Yan [23]	2020	44	288.7	54.3	0	0	0
Chao Huang [22]	2020	32	185.81	26	0	0	0

The literature shows that the application of barbed thread in laparoscopic surgery and robotic surgery can significantly shorten the suture time under the laparoscope [11, 24–29]. The relevant experience necessary for performing hand-sewn esophageal jejunostomy and the suture technique has been extensively discussed in the literature [15, 22, 23, 30, 31]. The research experience of the team revealed the following: (1) The posterior wall of the anastomotic stoma was sutured continuously with barbed sutures, and the entire posterior wall was sutured intermittently with varus sutures; the anterior wall of the anastomotic site was first sutured with interrupted eversion; then, the seromuscular layer was continuously sutured with barbed sutures. This suturing method is relatively simple to perform and easy to promote, and blood supply is guaranteed, avoiding the inconvenience of inserting the anvil of the circular stapler and the excessive free range of the linear stapler to the esophageal stump, and the advantages of circular and linear staplers in TLTG are integrated. (2) After continuous suture of the posterior wall of esophageal stump and the posterior wall of jejunum, the barbed wire is retained, which is easy to expose the operation area of anastomosis by pulling the barbed wire, and it can be used for subsequent anastomosis. In addition, because the esophagus and jejunum are fixed together before the anastomosis, the problem of short esophageal stump, high tension, and even retraction of the thoracic cavity can be solved for flat people with high tumor position. (3) The occurrence of anastomotic stenosis and bleeding could be effectively reduced by performing a skillful fine suture operation and selecting the appropriate opening diameter to avoid excessive varus suturing. No anastomotic complications occurred even if edema of the wall and small diameter and high position of the lesion were found during the operation. (4) Hand-sewn anastomosis is also convenient for cutting the cut edge of the esophageal stump. The stump can be cut with scissors under laparoscopic guidance, and then removed with spoon forceps, and the distance between the cutting edge and the lesion was≥3cm. For smaller lesions, preoperative endoscopic silver clip marking or intraoperative endoscopic positioning is needed. If the intraoperative pathological result was negative, an anastomosis was performed. A hole was punched in the stump of the esophagus with an ultrasonic knife, and the digestive juice was fully attracted. Then, the small mouth was sutured, and the posterior wall of the esophagus and the posterior jejunum were fixed and sutured, which can effectively avoid the overflow of digestive juice. (5) Surgeons who are inexperienced in hand-sewn anastomosis should be very cautious in choosing the high anastomosis. If they want to pass the learning curve, they can start from TLDG. After all, the safety of patients is still our most fundamental pursuit.

The limitation of this study is that it is a retrospective study in a single institution. The patients were not from the same period of time and were not randomized, and the sample size was small. Also, there might be selection bias because we were more inclined to perform hand-sewn anastomosis for some patients with lesions at relatively high positions and with a greater BMI. We recommend clinical verification of these results with prospective randomized controlled trials conducted with large sample sizes.

## Conclusion

The findings of this study suggest that hand-sewn anastomosis is a safe and feasible surgical method. Under the conditions of gaining mastery of laparoscopic suture technology, and strictly adhering to the indications, hand-sewn anastomosis can be an effective supplement to instrument anastomosis in TLTG, and offers an effective alternative technology for cases of local special tissues in the esophageal stump. It may be more convenient regarding obesity, a relatively high position of the anastomosis, edema of the esophageal wall, and short jejunal mesentery.

## Data Availability

Not applicable

## Abbreviations

BMI: Body mass index
LATG: Laparoscopic-assisted total gastrectomy
LDG: Laparoscopic distal gastrectomy
TLTG: Totally laparoscopic total gastrectomy
